# Brachial Plexus Chemical Neurolysis with Ethanol for Cancer Pain

**DOI:** 10.1155/2018/8628645

**Published:** 2018-07-24

**Authors:** Tuan-Hsing Loh, Samir Patel, Anish Mirchandani, Maxim Eckmann

**Affiliations:** Department of Anesthesiology, University of Texas Health Science Center at San Antonio (UTHSCSA), San Antonio, TX, USA

## Abstract

Chemical neurolytic nerve blocks have been successfully used to treat a variety of cancer-related pain. However, the literature has been sparse regarding neurolysis of the brachial plexus for cancer pain. We present a unique case report of a successful chemical neurolysis of the brachial plexus with dehydrated ethanol for a patient suffering from metastatic mammary carcinoma with tumor invasion of the right brachial plexus.

## 1. Introduction

Cancer pain can be difficult to manage. It is not unusual to incorporate interventional pain procedures to achieve satisfactory pain control in conjunction with pharmacologic treatment. Chemical neurolysis is a commonly used and versatile tool in cancer pain management. Target sites for chemical neurolysis may include intrathecal injections, superior hypogastric plexus, celiac plexus, lumbar sympathetic chain, ganglion impar, and peripheral nerves [1, 2]. In addition to pain relief, chemical neurolysis may also be used to manage regional spasticity [3, 4]. Common agents used for chemical neurolysis include ethanol 50–100% and phenol 5–10% [5]. The literature regarding the application chemical neurolysis to the brachial plexus has been sparse. We present a unique case report of the successful chemical neurolysis of the brachial plexus for the control of pain related to tumor invasion of the brachial plexus.

## 2. Case Presentation

A 59-year-old female initially presented to the emergency department 10 months prior with right arm pain and swelling. A computed tomography of the neck with contrast at the time showed two confluent masses in the right axillary and right supraclavicular regions encasing the right subclavian and axillary vein, the internal mammary artery, and narrowing of the lower internal jugular vein. Patient was subsequently diagnosed with primary mammary carcinoma of the axilla with metastasis. The patient was evaluated by the oncology and radiation oncology services and underwent multiple rounds of chemotherapy and radiation therapy. Her course of chemotherapy was complicated by thrombocytopenia and metastatic disease progression. The patient was determined to be a nonsurgical candidate. Gradually, her pain of the right upper extremity worsened, and the patient was started on opioid therapy. Despite titration of her oral medications to extended release morphine 90 mg two times a day, immediate release morphine 30 mg every 2 to 3 hours, methadone 5 mg daily, and gabapentin 800 mg three times daily, her pain control remained suboptimal. She was referred to our pain clinic for further management of her intractable pain.

**Figure 1:**
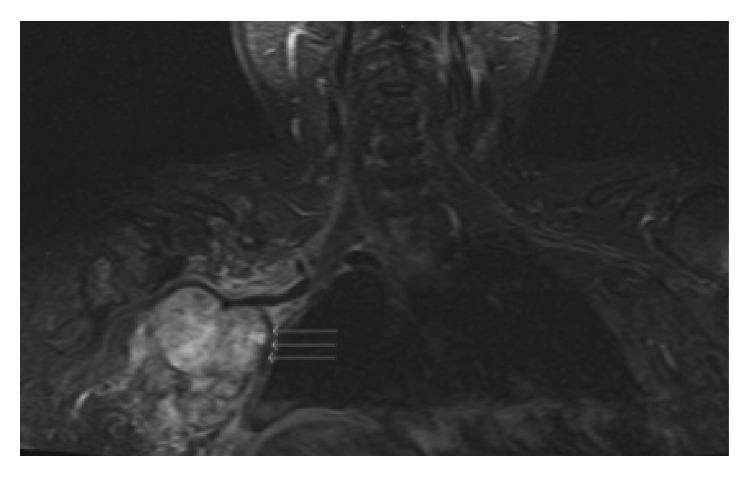
Tumor invasion of the right supraclavicular area.

On presentation, the patient reported a constant 10/10 pain on the numeric pain rating scale (NRS) of the right proximal humerus, right anterior and posterior shoulder, and right supraclavicular region. The pain was reported as dull, aching, burning, and electric in nature. Her pain was worsened by passive and active range of motion, and the pain at its best was a 7/10 with oral medications. Patient also reported progressive weakness of the entire right upper extremity. Magnetic resonance imaging of the brachial plexus was obtained, and the study revealed a mass encasing the right brachial plexus at the level of the divisions and cords as well as the right brachial artery (Figure 1).

Diagnostic brachial plexus block was performed in the hospital due to functional decline and acute worsening of pain. The brachial plexus was unable to be visualized using ultrasonography in the classic supraclavicular area due to the tumor effect, thus the block was performed at the level of the trunks using a lower interscalene approach. 18 mL of 0.5% ropivacaine was injected under direct ultrasound guidance. The patient reported complete pain relief lasting approximately 12 hours after the nerve block. Given the success of the diagnostic nerve block, the patient was offered the option of chemical neurolytic brachial plexus block with an extensive discussion of the unique risks, benefits, and alternatives. The patient elected to proceed to a right brachial plexus nerve block with dehydrated ethanol.

## 3. Procedure

**Figure 2:**
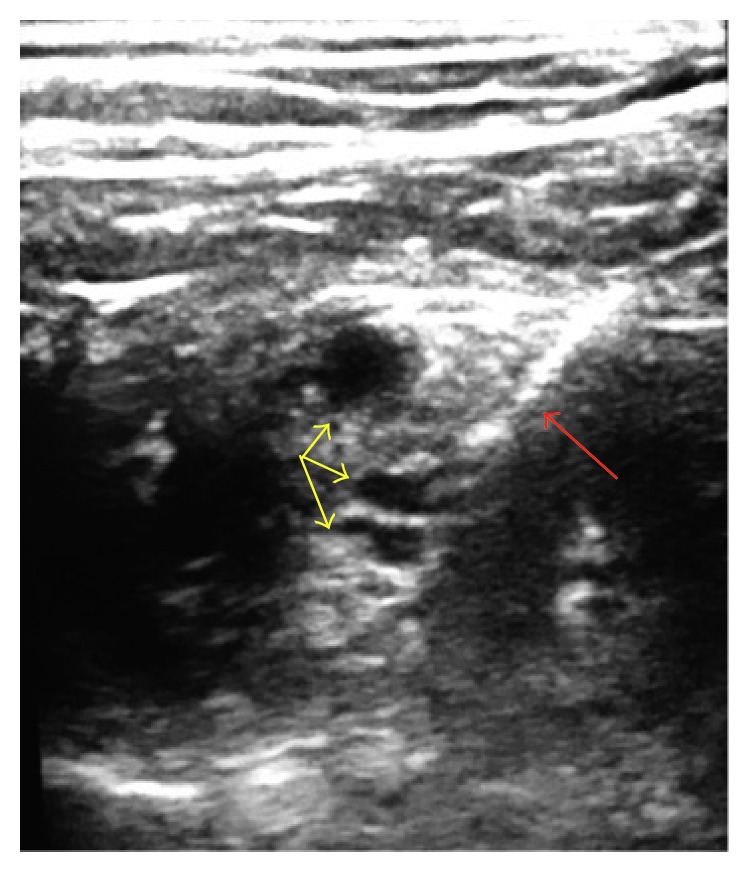
Chemical neurolysis of the right brachial plexus under ultrasound guidance. Red arrow: echogenic needle. Yellow arrows: brachial plexus at the level of the trunks.

The patient was positioned supine with the head of the bed elevated slightly and the head turned to the left. A linear high frequency ultrasound probe was used to identify the right brachial plexus at the level of the trunks (Figure 2). A 22-gauge 50 mm insulated echogenic needle (Braun, STIMU32250) was advanced in-plane under ultrasound guidance towards the brachial plexus. 7 mL of 1.5% mepivacaine with 1 : 200000 epinephrine was first injected to anesthetize the brachial plexus and surrounding structures. Then 11 mL of 90% dehydrated ethanol was injected. The needle was redirected during the procedure to ensure adequate spread of both the local anesthetic as well as the dehydrated ethanol.

The patient tolerated the procedure well. There were no immediate complications associated with the procedure. The patient's pain initially worsened for 3 days after procedure; however at 4 days after procedure, her pain was reduced to a tolerable level of 6/10 on the NRS scale, and the patient was subsequently discharged to a nursing home with hospice. On follow-up 10 days after procedure, the patient continued to report pain relief distal to the injection site and lack of motor strength in the right arm. However, she did notice pain proximal to the level of the procedure that was managed with her oral medications. Unfortunately, shortly after this follow-up encounter, the patient passed away due to progression of her metastatic disease.

## 4. Discussion

Cancer-related pain can be very difficult to manage, and, in certain cases, the pain can be intractable as seen in patients with tumor invasion of the brachial plexus. Effective brachial plexus blocks using a combination of local anesthetic and steroid for cancer pain have been described in the literature. However, the description of chemical neurolysis of the upper brachial plexus as a palliative treatment for the relief of cancer-related pain has been sparse with only a single documented phenol injection of the infraclavicular brachial plexus [6, 7]. Here we describe a unique case of successful chemical neurolysis using ethanol of the brachial plexus for the relief of cancer-related pain.

There are several factors that must be considered prior to performing a chemical neurolysis of the brachial plexus. Proper patient selection is essential due to the unique consequences of the procedure. Neurolytic blockade should only be performed for severe pain that is well localized and unresponsive to pharmacologic management with previously demonstrated positive diagnostic blocks [1, 8]. Permanent loss of motor and sensory function in the upper extremity is expected and should be thoroughly discussed with the patient. In circumstances when patients experience progressive functional decline and shortened life expectancy, it is appropriate for the clinical goal of treatment to be focused on achieving palliative pain reduction. Other possible complications from a brachial plexus chemical neurolysis include pneumothorax, ipsilateral Horner's syndrome, phrenic nerve damage, intravenous or intra-arterial injection, neuraxial injection, and quadriplegia. In the case presented, after a thorough discussion of these risks, the patient elected to pursue chemical neurolysis for improved comfort.

The choice of neurolytic agent is also an important consideration with specific implications. The most commonly used neurolytic agents are ethanol and phenol. The concentration of ethanol typically ranges from 50 to 100%, and the concentration of phenol typically ranges from 5 to 10%. Neurolysis with ethanol is associated with severe pain on injection. Ethanol neurolysis, therefore, is typically combined with a local anesthetic to attenuate the initial painful stimulus. Ethanol neurolysis is also associated with a higher incidence of neuritis when compared to phenol, and ethanol-induced neuritis may last from days to weeks [2]. Phenol is not associated with painful sensation on injection as it initially acts as a local anesthetic following injection. Studies have shown no significant difference in efficacy of neurolysis between ethanol and phenol [9]. However, axonal regeneration occurs sooner with phenol when compared to ethanol, which may result in a shorter duration of clinical effect [10].

The patient we presented also had thrombocytopenia with a platelet count of 52,000 on the day of the procedure, which increased her risk of bleeding. However, the benefits of the procedure outweighed the risks as the thrombocytopenia was stable, and the patient exhibited no active signs of bleeding. The patient experienced postprocedural neuritis that gradually subsided in intensity. The residual pain that the patient experienced around the neck area may be explained by local tumor invasion rather than residual neuritis. We highlight a lower interscalene approach as opposed to a traditional supraclavicular approach due to the difficulty with nerve visualization from tumor invasion. The patient we present is a unique case, which outlines the clinical complexities of treating cancer-related pain and the considerations involved for the use of chemical neurolysis of the brachial plexus as a palliative pain treatment option.

## 5. Conclusion

Chemical neurolysis of the brachial plexus with ethanol is a reasonable option for the treatment of cancer-related pain in appropriately selected patients.
